# Effects of Organic Montmorillonite (OMMT) and Pre-Orientation on Property of Poly(l-lactic acid) (PLLA)/Ethylene Propylene Diene Monomer (EPDM) Blends

**DOI:** 10.3390/polym12010106

**Published:** 2020-01-04

**Authors:** Di Song, Kai Wang, Jianing Shen, Long Zhao, Nai Xu, Sujuan Pang, Lisha Pan

**Affiliations:** 1 School of Materials Science and Engineering, Hainan University, Haikou 570228, China; songdi716@163.com (D.S.); wang_kai126@163.com (K.W.); shenjianing0823@163.com (J.S.); m18334680283@163.com (L.Z.); 2 Hainan Provincial Fine Chemical Engineering Research Center, Hainan University, Haikou 570228, China; psjuan@hainanu.edu.cn (S.P.); happylisap@163.com (L.P.); 3 School of Science, Hainan University, Haikou 570228, China; 4 School of Chemical Engineering and Technology, Hainan University, Haikou 570228, China

**Keywords:** poly(l-lactic acid), ethylene propylene diene monomer, montmorillonite, pre-orientation, calendering, tensile performance

## Abstract

Poly(l-lactic acid)/ethylene propylene diene monomer/organic montmorillonite (PLLA/EPDM/OMMT) samples were melt-compounded and then processed into sheets via two routes, namely, compression-molding and calendering. Tensile performance, morphology, and thermal property of the samples were investigated. Tensile test showed that the incorporation of OMMT resulted in significant enhancement in the tensile ductility of the PLLA/EPDM samples. SEM observation revealed that EPDM domain size decreased largely with increasing OMMT loading, indicating the compatibility of OMMT with PLLA/EPDM blends. Moreover, the elongation at break, tensile yield strength, and modulus of the calendered samples were found to be much higher than those of the compression-molded samples. It can be attributed to the pre-oriented rigid amorphous fraction of PLLA matrix and pre-stretched EPDM phases in the calendered samples produced by the stretching/shearing effect of calendering. Compared to the spherical/ellipsoidal EPDM particles in the compression-molded samples, these stretched EPDM phases with higher aspect ratio in the calendered samples can be more effective to initiate craze, and terminate the craze growing to a crack along transversal direction. Therefore, the calendered samples show a better tensile ductility than the compression-molded ones. Moreover, annealing was carried out to increase the crystallinity of the samples. Tensile performance, morphology, and thermal property of the annealed samples were also systematically investigated.

## 1. Introduction

Poly(l-lactic acid) (PLLA) is a synthetic polymer obtained by direct polycondensation of lactic acid or ring-opening polymerization of lactide [1,2,3]. At present, it has become one of the most widely used commercial bio-based polymers due to its versatile nature and fascinating properties, such as high mechanical strength, good biodegradability, and renewability [4,5,6]. Therefore, PLLA has been attracting progressively more interest as a potential alternative to some petroleum-based general purpose plastics that are non-degradable [7]. Nevertheless, its inherent brittleness is a major imperfection that restricts the application of PLLA to a larger extent and scope, thus numerous approaches have been adopted to improve the toughness and ductility of PLLA [8,9,10].

Melt blending of PLLA with some highly elastic or flexible polymers is a common toughening modification method. Polymers as toughening phases mainly include biodegradable polymers, such as poly(butylene adipate–*co*–terephthalate) (PBAT) [11], poly(butylene succinate–*co*–adipate) (PBSA) [12], poly(ε-caprolactone) (PCL) [13]; and non-degradable polymers, such as polyethylene (PE) [14], poly(ethylene vinyl acetate) (EVA) [15], ethylene propylene diene monomer (EPDM) [16], thermoplastic polyurethane (TPU) [17], and natural rubber (NR) [18], etc. However, the compatibility between PLLA and the toughening polymers is usually not good, thus some new compatibilizers have to be introduced to improve the compatibility of the PLLA blends.

The common compatibilizers for the immiscible PLLA blends can be divided into the following two categories: reactive compatibilizers and non-reactive compatibilizers. Among them, reactive compatibilizers mainly include organic peroxides, organic epoxides, polyisocyanates, etc. [19,20,21,22]. The compatibilizing effect of the reactive compatibilizers is usually very strong; however, the reaction process is difficult to control and might lead to overreaction or even cross-linking, which leads to significant change in the rheological behavior of PLLA and decrease in its processability. Non-reactive compatibilizers mainly include amphiphilic polymers such as block copolymers [23,24], graft copolymers [25,26], and nano-inorganic particles [16,27,28,29,30,31], etc. In general, nano-inorganic particles can be used as functional fillers to improve the physical performances of the PLLA blends, such as strength, modulus, conductivity, and flame retardancy, etc. However, in some cases, researchers have found that the nano-inorganic particles have a compatibilization effect to enhance the interfacial adhesion and/or reduce the domain size of the dispersed phase due to its selective distribution at the interface of the PLLA blends. For a polymer A/polymer B/nano-inorganic particles blending system, selective distribution of the nanoparticles (NPs) can be decided by the thermodynamic or kinetic factors [32,33,34,35,36]. At present, nano-inorganic particles used as compatibilizers in the PLLA blends mainly include organic montmorillonite (OMMT), carbon nanotubes, nano-silica, and functionalized polyhedral oligomeric silsesquioxane (POSS) [16,27,28,29,30,31]. For some PLLA blending system compatibilized by the nano-inorganic particles [16,28,29,30,31], the interfacial adhesion enhances and/or the size of dispersed phase domain decreases greatly with the incorporation of nano-inorganic particles, thus leading to an improvement in toughness and ductility of the PLLA blends. In our previous research [16], the PLLA/EPDM/OMMT samples were fabricated by compression-molding. Scanning electron microscopy (SEM) analysis indicated that all the PLLA/EPDM/OMMT samples prepared by compression-molding showed a typical immiscible dispersed droplet morphology. With increasing OMMT loading, the size of the spherical/ellipsoidal EPDM particles decreased significantly. It was confirmed from transmission electron microscopy (TEM) observation that a part of OMMT particles was selectively located at the PLLA/EPDM interface, leading to a great decrease in the EPDM particles size. Under a given rubber content, the smaller the rubber particles, the stronger was their ability to induce the matrix yielding and terminate the development of crazes. With incorporation of 1 phr OMMT, the PLLA/EPDM/OMMT sample exhibited a significant increase in toughness and ductility. However, with the addition of excessive amount of OMMT into the PLLA/EPDM blends, the toughness and ductility of the samples decreased obviously. It could be attributed to overloading of OMMT that might have induced an aggregation of OMMT particles in the matrix. That could result in a premature rupture behavior of the samples.

Up to date, many literature studies have reported melt compounding of the PLLA blends compatibilized with nano-inorganic particles by compression-molding or injection-molding. Nevertheless, the effect of calendering processing on the microstructures and properties of the PLLA/EPDM blend compatibilized by nano-inorganic particles has rarely been reported. In this study, PLLA/EPDM samples with different amounts of OMMT were fabricated through melt extrusion and then processing via calendering. The influences of stretching/shearing effect of calendering as well as OMMT on tensile performance, morphology, and crystalline structure were systematically investigated. For comparative analysis, the compression-molded PLLA/EPDM/OMMT samples were also prepared by a “melt extrusion-compression molding” process. Furthermore, annealing was carried out to increase the crystallinity of the samples. Finally, the performance comparison of the annealed samples was also analyzed and discussed.

## 2. Materials and Methods 

### 2.1. Materials and Processing

Semi-crystalline grade PLLA resin (4032D) was purchased from Nature Works (Minnetonka, MN, USA). It contained about 2% D-lactide units with a density of 1.25 g cm^−3^. EPDM pellets (3722P) with a density of 0.86 g cm^−3^ were purchased from Dow Chemical Company (Midland, MI, USA). OMMT powder (1.44PSS) was purchased from NANOCOR (Arlington Heights, IL, USA), for which organic intercalating agent was bis(hydrogenated tallow) dimethylammonium/siloxane. 

PLLA and EPDM pellets were dried in a vacuum oven at 45 °C for 6 h. OMMT was dried in an air oven at 80 °C for 5 h. Then, the neat PLLA and PLLA/EPDM/OMMT (90/10/*x*) blends were prepared using a co-rotating twin screw extruder (SHJ-20, Nanjing Giant Co., Ltd., Nanjing, China). The extruder screw had a diameter of 20 mm and L/D of 40. The temperatures from hopper to die were set at 160, 200, 200, 200, 200, and 200 °C, respectively. Furthermore, the rotating speed of twin screw was fixed at 120 rpm. The extrudate was cooled by water and then cut using a pelletizer to obtain blended pellets. Finally, the blended pellets were dried to remove moisture before further processing.

In this study, the obtained blends are referred to as PLLA/EPDM/OMMT (90/10/*x*), where 90/10 denotes weight ratio of PLLA/EPDM, and *x* denotes OMMT parts per hundreds of total resion (phr). Herein, *x* = 0, 1, 2, and 4 phr, respectively. 

**Figure 1:**
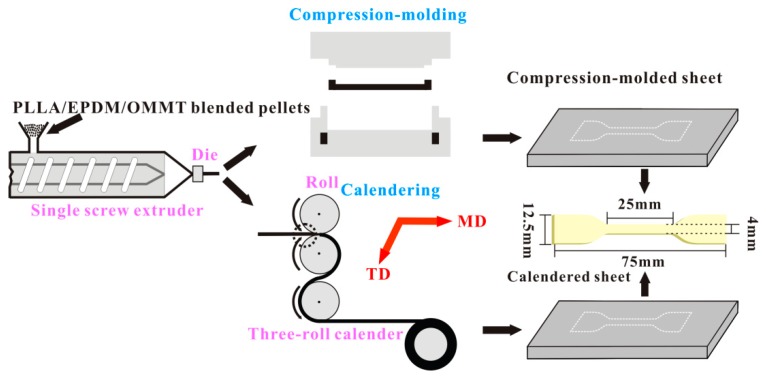
Schematic illustration of calendering process and compression–molding process.

Preparation of calendered samples: The blended pellets were then extruded using a single screw extruder (LSJ-20, Shanghai Kechuang Plastic Machinery Factory, Shanghai, China) with a slit die (H × W, 0.90 mm × 45.50 mm). The extruder screw had a diameter of 20 mm and L/D of 25. The temperatures from hopper to die were set at 140, 210, 210, and 210 °C, respectively. The single screw speed was fixed at 20 rpm. Then, the extruders were immediately stretched and calendered using a three roller calender to obtain continuous belt-like samples. By adjusting the roll distance and rolling speed, the thickness and draw ratio (DR) of the obtained belt-like samples were kept at 0.35 ± 0.05 mm and 5.5 ± 0.2, respectively. The schematic illustration of the extruding-calendering process is shown in Figure 1. The belt-like samples were cut into dumbbell specimens along machine direct (MD) for tensile test.

In order to obtain fully crystallized calendered samples, some belt-like samples were annealed using a hot press with a preload of 1 MPa. After being held in the hot press at 80 °C for 45 min, the annealed calendered samples were obtained. The annealed belt-like samples were also cut into dumbbell samples along MD for the tensile test.

Preparation of compression-molded samples: The blended pellets were extruded using a single screw extruder, similar to that in the extrusion process discussed above. Furthermore, the extrudates were directly collected and pressed into blanks with suitable weight. The pre-pressed blanks were compression-molded using a hot press at 210 °C and 15 MPa for 8 min. Then, the closed mold with in-mold melt were quickly transferred to a water cooling system with clamping pressure of 15 MPa and cooled down to room temperature at a rate of around 100 °C min^−1^. As a result, the compression-molded sheets with thickness of 0.35 mm were obtained. The schematic illustration of the extruding-compression molding process is also shown in Figure 1. The compression-molded sheets were cut into dumbbell specimens for the tensile test.

In order to obtain fully crystallized compression-molded samples, a part of each compression-molded sheet was annealed using a hot press with a preload of 1 MPa. After being held in the hot press at 80 °C for 45 min, the annealed compression-molded sheets were obtained. The annealed sheets were also cut into dumbbell specimens for tensile tests.

### 2.2. Characterization

#### 2.2.1. Tensile Performance

Tensile yield strength and elongation at break of the samples were measured using a universal tensile testing machine (WDW-1, Jinan Yi Nuo Century Test Instrument Co., Ltd., Jinan, China) at a cross-head speed of 25 mm min^−1^. Moreover, tensile modulus of the samples was measured at a cross-head speed of 5 mm min^−1^. The samples were stored for 72 h at room temperature before testing. The results presented are an average of five valid tests per sample with standard deviation.

#### 2.2.2. Scanning Electron Microscopy

Scanning electron microscopy (SEM, S-3500N, Hitachi, Tokyo, Japan) was conducted in order to research the morphology of the samples. Samples were previously cryo-fractured after immersion in liquid nitrogen for 5 min. In addition, in order to more clearly observe the dispersed phase morphology, the cryo-fractured surfaces of the blend samples were extracted with cyclohexane at ambient temperature for 12 h to selectively etch and remove the EPDM phase. All the cryo-fractures of the samples were sputter coated with gold prior to scanning. An acceleration voltage of 10 kV was used for all observations.

#### 2.2.3. Differential Scanning Calorimetry

Standard differential scanning calorimetry (DSC) measurements were carried out using a DSC instrument (Q100, TA Instruments, New Castle, DE, USA) to record heating DSC thermograms and obtain crystallinity ($X_{c}$) of the samples. DSC thermograms were recorded upon heating from 30 to 200 °C at a heating rate of 10 °C min^−1^ in N_2_ atmosphere. Moreover, the related thermal parameters (i.e., cold crystallization exothermic enthalpy ($\Delta H_{\mathrm{cc}}$), α´-to-α phase transition exothermic enthalpy ($\Delta H_{\mathrm{cc}}^{*})$, and melting endothermic enthalpy (${\Delta H}_{m}$)) could be obtained from the DSC traces.

*X*_c_ of the samples can be calculated by using Equation (1) as follows:

(1) $$X_{c}=\frac{\left|\Delta H_{m}\right|-\left|\Delta H_{\mathrm{cc}}\right|-\left|\Delta H_{\mathrm{cc}}^{*}\right|}{\omega_{\mathrm{PLLA}}\left|\Delta H_{m}^{0}\right|}\times 100\%$$

where ω_PLLA_ is mass fraction of PLLA in the specimen, $\Delta H_{m}^{0}$ is ideal melting enthalpy of the 100% crystalline PLLA (−93.70 J g^−1^) [37]. In this study, the endothermic enthalpy is defined as negative in the DSC measurements.

#### 2.2.4. X-Ray Diffraction

X-ray diffraction (XRD, Bruker D8 ADVANCE, Karlsruhe, Germany) was employed to evaluate the crystalline structure of PLLA matrix. The Cu Kα radiation (λ = 1.54 Å) was operated at 40 kV and 40 mA. XRD data were recorded in the range of 5° ≤ 2θ ≤ 35° with a scan speed of 2 °/min. Moreover, the intercalated structure of OMMT dispersed in the samples was also characterized by XRD in the range of 1° ≤ 2θ ≤ 10° with a scan speed of 2 °/min.

## 3. Results and Discussion

### 3.1. Properties of PLLA/EPDM/OMMT (90/10/x) Samples 

#### 3.1.1. Tensile Properties

**Figure 2:**
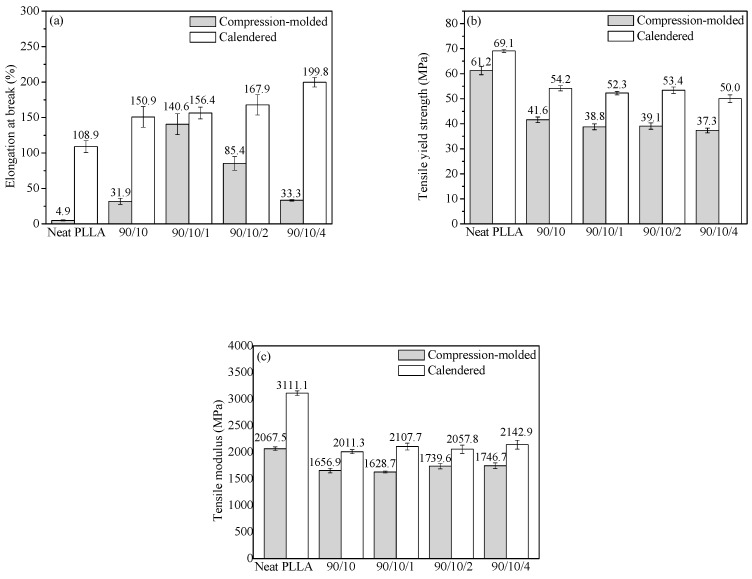
(**a**) elongation at break, (**b**) tensile yield strength, and (**c**) tensile modulus of PLLA and PLLA/EPDM/OMMT (90/10/*x*) samples prepared by compression-molding and calendering.

The tensile performance of the PLLA/EPDM/OMMT (90/10/*x*) samples prepared by calendering is presented in Figure 2. For comparative analysis, the tensile performance of the ones prepared by compression-molding is also shown in Figure 2. Moreover, Figure S1 (in Supplementary Materials) shows the tensile stress–strain curves of all the samples.

Figure 2a exhibits that the elongation at break (*ε*) of the compression-molded PLLA sample is very low (about 4.9%). With the incorporation of EPDM, the compression-molded PLLA/EPDM (90/10) sample shows an improvement in tensile ductility (*ε* = 31.9%). Moreover, the tensile ductility of the compression-molded PLLA/EPDM/OMMT (90/10/x) samples varies significantly with different OMMT loadings. With the addition of a small amount of OMMT into the PLLA/EPDM blends, the tensile ductility of the compression-molded ternary sample increases significantly. Compared to the compression-molded PLLA/EPDM (90/10) sample, *ε* of the compression-molded PLLA/EPDM/OMMT (90/10/1) sample increases by almost fourfold (*ε* = 140.6%). As reported in our previous research [16], the compatibilizing effect of OMMT in the immiscible PLLA/EPDM blend, resulting from the selective location of OMMT nanoparticles at the interface of PLLA/EPDM, should be responsible for the enhancement in tensile ductility of the PLLA/EPDM/OMMT samples. With the further increase of OMMT loading, an obvious decrease in *ε* is observed as presented in Figure 2a. For example, *ε* decreases to 85.4% for the compression-molded PLLA/EPDM/OMMT (90/10/2) samples and further reduces to 33.3% for the compression-molded PLLA/EPDM/OMMT (90/10/4) samples. This could be attributed to overloading of OMMT that may induce an aggregation of OMMT particles in the matrix leading to a premature rupture behavior of the samples. Furthermore, both the tensile yield strength and modulus of the compression-molded PLLA/EPDM (90/10) sample decrease to some extent with incorporation of EPDM. Notably, with the increase in the OMMT loading from zero to 4 phr, a very mild decrease in tensile yield strength and a slight increase in tensile modulus are found for the compression-molded PLLA/EPDM/OMMT samples. 

Furthermore, Figure 2 demonstrates that the calendered samples show better tensile performances than the compression-molded samples with the same component ratio. Figure 2a shows that compared to the compression-molded PLLA sample with low ductility (*ε* = 4.9%), the calendered PLLA sample acquires an unexpected improvement of tensile ductility, i.e., *ε* = 108.9%. During the calendering processing using the tree roller calender, the extruded PLLA melt gets stretched and calendered, and finally solidifies with a rapid cooling rate. The stretching/shearing effect imposed by the three rollers calender can induce amount of PLLA segments to orient along machine direct (MD) to some extent. Moreover, these oriented PLLA segments can form some pre-oriented amorphous structures (i.e., rigid amorphous fraction, RAF [38]) in the PLLA matrix. The pre-oriented RAF in the longitudinal direction of the tensile specimens can act as stress concentration points to induce shear yielding of the PLLA matrix, while the specimen is drawn in a tensile test at room temperature. As a result, the calendered PLLA sample shows an improved tensile ductility. The respective tensile fracture mechanisms for the neat PLLA samples prepared by the different forming methods will be proposed and discussed in Section 3.1.3.

With the addition of 10 wt. % EPDM into PLLA matrix, *ε* of the calendered PLLA/EPDM (90/10) sample reaches to 150.9%. Moreover, with the increase of OMMT loading, the tensile ductility of the calendered PLLA/EPDM/OMMT (90/10/*x*) is further improved. With the increase in the OMMT loading from 1 to 4 phr, *ε* of the calendered ternary sample increases from 156.4 to 199.8%. For the compression-molded ternary samples, upon addition of 1 phr OMMT into the PLLA/EPDM blend, *ε* reaches a maximum of 140.6%. Nevertheless, with a further increase in OMMT loading, the tensile ductility of the compression-molded ternary samples shows a drastic drop (85.4% at 2 phr of OMMT, and 33.3% at 4 phr of OMMT, respectively). The agglomeration of OMMT particles is the plausible reason for the decreased ductility of the compression-molded samples with relatively high OMMT loading. Different from the premature rupture behavior of the compression-molded samples with higher OMMT loading, the tensile ductility of the calendered ternary samples increases rapidly with increasing OMMT loading from zero to 4 phr. Next, in order to better analyze and understand the different tensile fracture behaviors of the compression-molded samples and the calendered ones, the phase morphology of the samples was studied by SEM. 

Furthermore, compared to those of the compression-molded samples, the tensile yield strength and the tensile modulus of the calendered PLLA/EPDM/OMMT samples are enhanced largely, as shown in Figure 2. The stretching/shearing effect of the three rollers calender is responsible for the formation of pre-oriented RAF in the PLLA matrix, which may significantly enhance the tensile performances of the samples. For example, the tensile yield strength and modulus of the compression-molded sample containing 4 phr OMMT are 37.3 and 1746.7 MPa, respectively. Comparatively, the tensile yield strength and modulus of the calendered one containing 4 phr OMMT increase to 50.0 and 2142.9 MPa, respectively.

#### 3.1.2. Microscopic Morphology Analysis 

**Figure 3:**
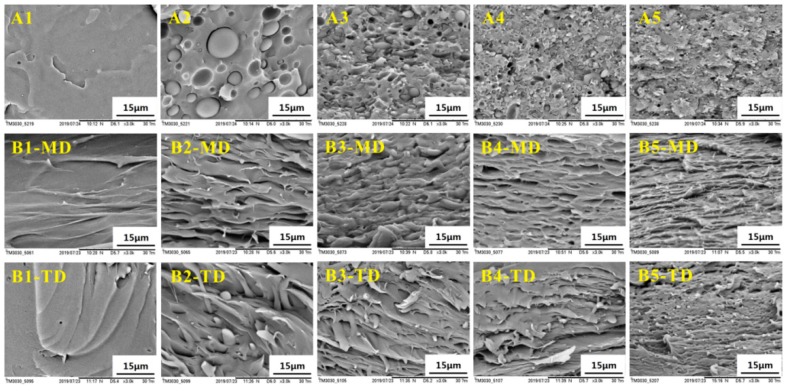
SEM images of PLLA and PLLA/EPDM/OMMT (90/10/x) samples prepared by (**A**) compression-molding/(**B**) calendering (**1**-Neat PLLA, **2**-90/10, **3**-90/10/1, **4**-90/10/2, and **5**-90/10/4).

In order to study the morphologies of the PLLA/EPDM/OMMT samples obtained by different processing methods, the cryo-fractured surfaces of the samples were observed by SEM, as shown in Figure 3. Figure 3A1–A5 present the morphology of the compression-molded samples. Clearly, the cryo-fractured surface of the PLLA sample is fairly flat (see Figure 3A1). In case of the compression-molded PLLA/EPDM (90/10) sample, a typical immiscible dispersed droplet morphology with poor interfacial adhesion and large EPDM domains size is observed in Figure 3A2. Figure 3A3 demonstrates that, upon addition of 1 phr OMMT, the EPDM domain size decreases largely, and the PLLA/EPDM interface becomes blurred. With increasing OMMT loading to 2 and 4 phr, the EPDM domain becomes smaller with an extremely blurred interface (see Figure 3A4,A5). In order to take a clearer observation of the EPDM domain morphology, SEM images of cyclohexane-etch cryogenically fracture surfaces of the compression-molded blends are presented in Figure S2 (in Supplementary Materials). Based on the SEM images in Figure S2, 200 randomly selected EPDM particles for each blend sample were measured to obtain the average EPDM particle size ($\bar{d}$) by using a soft for particle size measurement and statistics (Nano Measurer V.1.2, Fudan University, Shanghai, China). The obtained EPDM particle diameter distribution and the average particle size ($\bar{d}$) are also presented in Figure S2. As shown in Figure S2, the average EPDM particle size ($\bar{d}$) drastically decreases from 5.05 to 0.82 μm with increasing OMMT loading from zero to 4 phr, indicating that OMMT has a strong compatibilization effect on the PLLA/EPDM bends. In our previous research [16], TEM was applied to research the distribution of OMMT in the PLLA/EPDM/OMMT blends. It was found that a large number of OMMT nanoparticles concentrated at interfacial region between PLLA and EPDM phases, as well as in the EPDM phase. The selective distribution of OMMT can be attributed to the fact that the OMMT nanoparticles had a higher affinity with an EPDM domain. The OMMT nanoparticles located at the PLLA/EPDM interface could reduce the interfacial tension and inhibit the coalescence of EPDM phase during blending. As a result, the EPDM domain size significantly decreases with the addition of OMMT. 

Figure 3(B1–MD)–(B5–MD) show SEM images of the cryo-fractured surfaces of the calendered samples in a longitudinal direction (along the machine direction, MD). Moreover, SEM images of the cryo-fractured surfaces of the calendered samples in transversal direction (TD, perpendicular to MD) are presented in Figure 3(B1–TD)–(B5–TD). In addition, SEM images of cyclohexane-etch cryogenically fracture surfaces of the calendered blend samples in longitudinal direction (along MD) are presented in Figure S3 (in Supplementary Materials). Compared to the spherical/ellipsoidal EPDM particles in the compression-molded samples, an amount of stretched EPDM phases in fibre/ribbon/slice forms appear in the calendered samples. With increasing OMMT loading from zero to 4 phr, the stretched EPDM domain becomes finer and more compact due to the compatibilization effect of OMMT. The strong stretching/shearing effect of three rollers calender on the extruded melt is responsible for the formation of the stretched EPDM domain along MD, as well as the formation of oriented PLLA RAF. In contrast to the calendering processing, the compression-molding process can impose only a very weak shearing effect on the melt. In this situation, most of the flow history of the melt in compression-molding could be relaxed rapidly before the melt cooled down and solidified. As a result, the PLLA/EPDM/OMMT samples prepared by the compression-molding technique can be viewed as an isotropic material without obvious oriented structures. The spherical/ellipsoidal EPDM particles dispersed in the compression-molded samples provide a strong evidence to support this view. 

**Figure 4:**
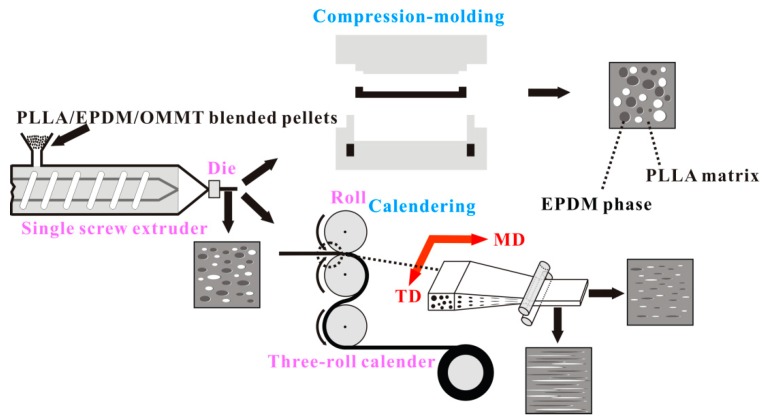
Comparison of morphology between PLLA/EPDM/OMMT samples prepared by compression-molding and calendering.

Figure 4 presents the proposed morphology evolution of the blending samples during the calendering processing and the compression-molding as well. 

#### 3.1.3. Crystallization Property

**Figure 5:**
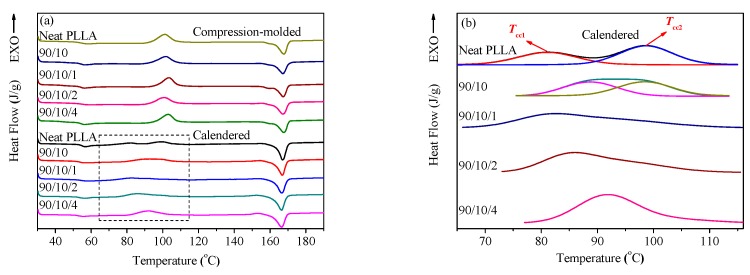
(**a**) DSC traces of PLLA and PLLA/EPDM/OMMT (90/10/*x*) samples prepared by compression-molding and calendering, and (**b**) DSC thermograms of calendered samples in the temperature range of 65–115 °C.

**Figure 6:**
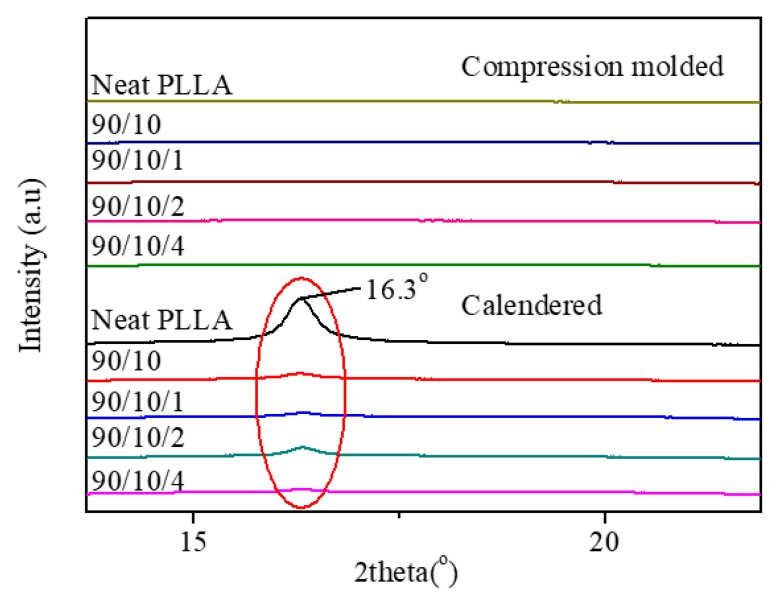
XRD patterns of PLLA and PLLA/EPDM/OMMT (90/10/*x*) samples prepared by compression-molding and calendering.

DSC and XRD measurements were performed to research the thermodynamic behavior and crystallization property of the calendered samples, and those of the compression-molded ones as well. Figure 5 shows the heating DSC traces of all the samples, from which the corresponding thermodynamic parameters are listed in Table S1 (in Supplementary Materials). Moreover, Figure 6 presents the XRD patterns of the samples in the range of 5° ≤ 2θ ≤ 35°. 

Figure 5a illustrates that there appears to be a cold crystallization peak in the temperature range of 100.9–103.5 °C for all the compression-molded samples (see Table S1), which indicates that the compression-molded samples exhibit a low crystallinity or even amorphous nature [39]. Furthermore, the parameters related to crystallinity of the samples were calculated according to Equation (1), and the corresponding results are listed in Table S1. As expected, the crystallinity of each compression-molded sample is very low (i.e., ≤0.55%), whose PLLA matrix is almost amorphous. This result is consistent with the finding obtained from the XRD patterns shown in Figure 6. The XRD diffractions of the compression-molded samples show a weak and wide dispersing diffraction peak, indicating their amorphous nature. The low crystallinity of the compression-molded samples can be attributed to the poor homogeneous nucleation and crystallization ability of the PLLA melt in a completely amorphous state. In contrast, the crystallinity of the calendered PLLA sample increases from 0.55% for the compression-molded PLLA sample to 11.59%. A similar moderate increase in crystallinity was also found in the calendered PLLA/EDPM/OMMT samples. This is attributed to the fact that the extruded melt was subjected to a strong stretching/shearing effect when passing through the three roller calender, which made an amount of PLLA segments orient along MD. Consequently, these oriented PLLA segments and their oriented RAF could act as crystal seeds to further induce crystallization of the PLLA melt before the sample cooled down and solidified by the chill roller [40]. However, the rapid cooling rate during calendering could not allow the full crystallization of the calendered samples. Therefore, the crystallinity of the calendered samples can only reach to around 4.14–11.59%. Apparently, the crystalline structure of the calendered samples is far from full growth. The similar conclusion can also be obtained from their XRD patterns (see Figure 6). Clearly, there is a weaker diffraction peak at 2θ = 16.3°, which indicates the formation of some underdeveloped crystals or mesophase in the samples. 

Moreover, compared to the single cold crystallization peak (100.9–103.5 °C) for the compression-molded PLLA sample, it is found from Figure 5 that the cold crystallization peak for the calendered PLLA sample splits into two peaks with weaker intensity at 81.1 and 98.8 °C, respectively. The lower cold crystallization peak ($T_{\mathrm{cc}1}$= 81.1 °C) corresponds to the crystallization of the oriented RAF and the recrystallization of the underdeveloped crystal, while the calendered PLLA sample is reheated during the heating DSC measurement. Moreover, the higher cold crystallization peak ($T_{\mathrm{cc}2}$ = 98.8 °C) may correspond to the cold crystallization of the unoriented PLLA amorphous fraction. When 10 phr of EPDM was added, $T_{\mathrm{cc}1}$ of the calendered PLLA/EPDM (90/10) sample increases up to 88.7 °C. With the addition of OMMT into the PLLA/EPDM (90/10) sample, the two cold crystallization peaks gradually overlap and merge into a wider peak. The incorporation of EPDM and OMMT shows a complex influence on the cold crystallization of the samples that needs further research in future. Nevertheless, owing to the existence of the pre-oriented RAF and underdeveloped crystal, the cold crystallization ability of the calendered samples could be enhanced, leading to the shift in their cold crystallization peak to lower temperature range compared to that of the compression-molded ones. 

According to the results obtained from the tensile test, SEM observation, XRD, and DSC measurements, the respective tensile fracture mechanisms for the calendered samples and the compression-molded samples were proposed as follows: *ε* of the compression-molded PLLA sample is only 4.9%. With the incorporation of 10 wt. % EPDM, *ε* of the compression-molded PLLA/EPDM (90/10) sample increases to 31.9%. Upon addition of 1phr OMMT into the PLLA/EPDM blend, *ε* of the compression-molded PLLA/EPDM/OMMT (90/10/1) sample increases significantly to 140.6%. Figure 3 shows that with the incorporation of 1 phr OMMT, the EPDM domain size decreases largely, and the PLLA/EPDM interface becomes blurred. According to the craze-shear band theory and Wu theory [41,42], the following facts can be highlighted: under a constant rubber content, the smaller the rubber particles, the stronger is their ability to induce the matrix yielding and terminate the development of crazes. Therefore, the compatibilizing effect of OMMT in the immiscible PLLA/EPDM blend should be responsible for the enhancement in tensile ductility of the PLLA/EPDM/OMMT samples. However, with the further increase in the OMMT loading, an obvious decrease in the tensile ductility is observed. This could be attributed to overloading of OMMT that may induce an aggregation of OMMT particles in the matrix leading to the early fracture of the samples.

**Figure 7:**
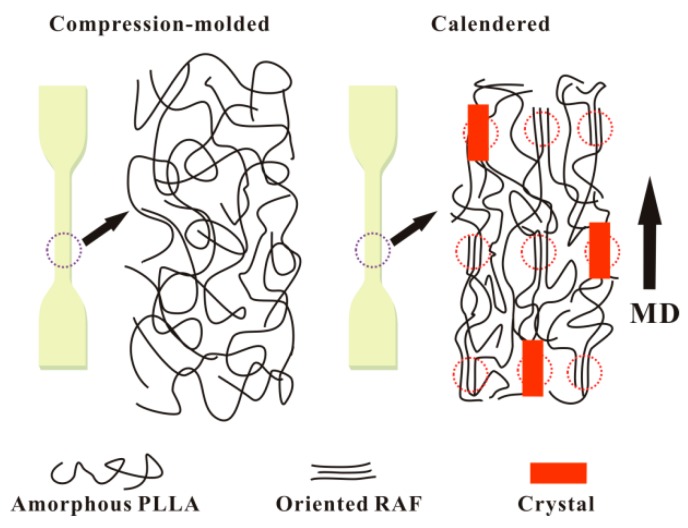
Schematic illustration of internal structures of neat PLLA samples prepared by compression-molding and calendering.

Compared to the low ductility (*ε* = 4.9%) of the compression-molded PLLA sample, *ε* of the calendered PLLA sample increases drastically to 108.9%. The brittle–ductile transition of the calendered PLLA sample maybe attributed to the fact that the pre-oriented structures (i.e., the RAF and the underdeveloped crystals) in the longitudinal direction can act as stress concentration points to induce shear yielding of the PLLA matrix, while the calendered PLLA sample is drawn in a tensile test at room temperature. The schematic illustration of internal structures of the neat PLLA samples prepared by compression-molding and calendering is presented in Figure 7.

**Figure 8:**
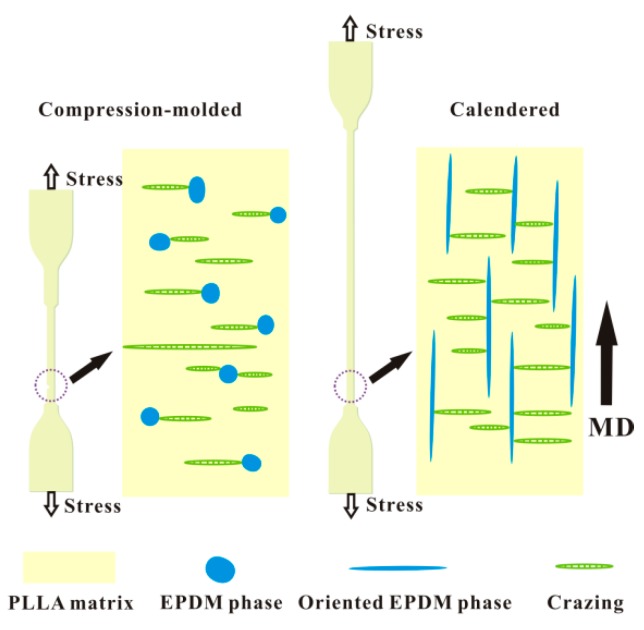
Schematic illustration of craze termination mechanism for PLLA/EPDM/OMMT (90/10/*x*) samples prepared by compression-molding and calendering.

With the addition of 10 wt. % EPDM, *ε* of the calendered PLLA/EPDM (90/10) sample increases to 150.9%. Moreover, with the increase of OMMT loading, the tensile ductility of the calendered PLLA/EPDM/OMMT (90/10/*x*) sample is further improved. With the increase in the OMMT loading from 1 to 4 phr, *ε* of the calendered ternary sample increases from 156.4 to 199.8%. Compared to the spherical/ellipsoidal EPDM particles dispersed in the compression-molded blending samples, the pre-stretched EPDM phases dispersed in the calendered blending samples show a higher aspect ratio. Therefore, the parallel and pre-stretched EPDM phase in longitudinal direction can be more effective to initiate craze; moreover, it can also be more effective to terminate the craze growing to a crack along transversal direction (see Figure 8). With the further increase of OMMT loading, the gradually narrowed pre-stretched EPDM phases further enhance the craze termination and yielding ability of the calendered ternary samples. As a result, the calendered ternary samples containing higher OMMT loading shows an enhanced tensile ductility because the pre-stretched EPDM could effectively stop the craze development, and even terminate the growth of micro-cracks resulting from the agglomeration of OMMT particles to avoid a premature rupture behavior of the materials. For example, compared to the decreased *ε* (33.3%) of the compression-molded one, *ε* of the calendered PLLA/EPDM/OMMT (90/10/4) sample reaches to a maximum value (*ε* = 199.8%), increasing by about 500%. Schematic illustration of craze termination mechanism for the PLLA/EPDM/OMMT samples prepared by compression-molding and calendering is shown in Figure 8.

#### 3.1.4. Intercalation Structure of OMMT

**Figure 9:**
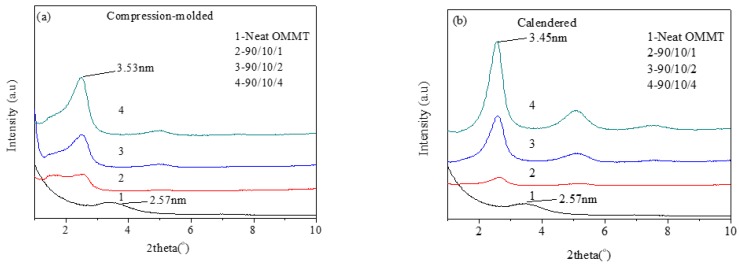
XRD patterns of PLLA and PLLA/EPDM/OMMT (90/10/*x*) samples prepared by (**a**) compression-molding and (**b**) calendering.

Figure 9 shows XRD patterns of OMMT powder and PLLA/EPDM/OMMT samples. The lamellar spacing of OMMT can be calculated from the 2θ data in the figure according to Bragg Equation (2) as follows:

(2) 2dsinθ=nλ,

where *d* represents the interlayer spacing, θ is the angle of the diffraction peak, and *λ* is the wavelength of the diffraction ray of Cu Kα (*λ* = 1.54 Å).

The first-order diffraction peak of OMMT powder is located at the position of 2θ = 3.43°. The interlayer spacing of OMMT powder is 2.57 nm obtained by using Equation (2). However, all the first-order diffraction peaks of the calendered and the compression-molded PLLA/EPDM/OMMT samples locate at 2θ = 2.55° ± 0.05°. The interlayer spacing of the PLLA/EPDM/OMMT samples ranges from 3.39 to 3.53 nm. This indicates the insertion of some polymer molecular chains into OMMT lamellae during blending and processing, thus leading to the formation of the intercalated nanocomposites [43]. The result also proves that there is no obvious difference between the OMMT intercalated structures in the compression-molded ternary samples and the calendered ones.

### 3.2. Properties of Annealed PLLA/EPDM/OMMT (90/10/x) Samples

Owing to the slow melt-crystallization rate of PLLA, the PLLA samples prepared by the common processing and forming methods have a very low crystallinity, and even become amorphous, similar to the compression-molded and calendered samples prepared in this study. A PLLA sample with low crystallinity shows some defects in its performance, such as poor durability, as well as weak resistance to thermal distortion. It is well known that increasing the crystallinity of PLLA sample is an effective method to improve its durability and heat resistance performance [9]. Next, the compression-molded and the calendered samples were annealed using a hot-press at 80 °C for 45 min in order to obtain the fully crystallized samples. The crystallization properties of the annealed samples were characterized by DSC and XRD measurements. Moreover, the microscopic morphology and tensile performance were also researched.

#### 3.2.1. Crystallization Property

**Figure 10:**
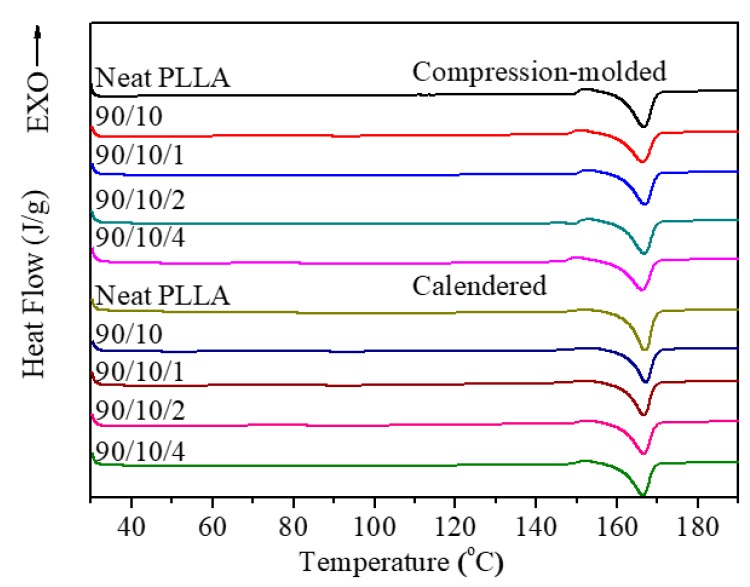
DSC traces of annealed PLLA and PLLA/EPDM/OMMT (90/10/x) samples prepared by compression-molding and calendering.

**Figure 11:**
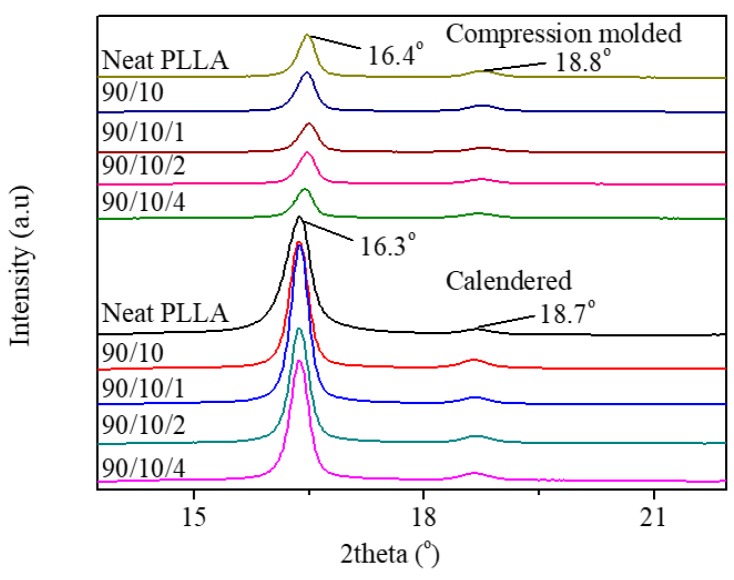
XRD patterns of annealed PLLA and PLLA/EPDM/OMMT (90/10/x) samples prepared by compression-molding and calendering.

DSC and XRD measurements were performed to research the thermodynamic behavior and crystallization property of the annealed samples. Figure 10 shows the heating DSC traces of all the annealed samples, from which the obtained corresponding thermodynamic parameters are listed in Table S2 (in Supplementary Materials). Figure 11 presents the XRD patterns of the annealed samples in the range of 5° ≤ 2θ ≤ 35°. 

Figure 10 shows the absence of cold crystallization peak in the heating DSC trace for all the annealed samples. Moreover, Table S2 summarizes that the crystallinity of all the annealed samples increases to more than 33.0%. This indicates full crystallization of these samples after annealing at 80 °C for 45 min. Li et al. also confirmed that the crystallinity of neat PLLA could increase to around 36% after the full cold crystallization of neat PLLA sample in an isothermal temperature ranging from 85 to 100 °C [44]. Table S2 presents that there is no obvious shift in *T*_m_ between these highly crystallized samples regardless of OMMT loading. Moreover, the appearance of a small exothermic peak ($T_{\mathrm{cc}}^{*}$) is observed in their heating DSC thermograms just prior to the melting peak. According to the report by Zhang et al. [45], this peak can be attributed to the α´-to-α phase transition with increasing temperature. 

Figure 11 demonstrates that all the annealed samples show two obvious diffraction peaks at 16.3°–16.4°and 18.7°–18.8°, corresponding to the reflections of (200/110) and (203) planes of the α’-form crystal [45]. This indicates that the XRD results are consistent with the DSC results. By annealing at 80 °C for 45 min, the highly crystallized compression-molded or calendered samples with α’-form crystals could be obtained successfully. 

Moreover, it is noteworthy that the diffraction intensities of the crystalline planes, in particular (200/110) planes of the annealed calendered samples, are sharper than those of the annealed compression-molded samples. Two main factors are responsible for the increase in the diffraction intensity of a semi-crystalline sample: (1) increase of crystallinity and (2) orientation of crystals [46,47,48]. Table S2 summarizes that there is no obvious difference between the crystallinity of the annealed calendered samples and the compression-molded samples. This indicates that the orientation of crystals may be responsible for the increase of the diffraction intensity. A plausible explanation is as follows: although the calendered samples were annealed at 80 °C in the hot press, the pre-orient RAF and the underdeveloped tiny crystalline grains could induce the cold crystallization of the PLLA matrix and transform them into the PLLA crystals in the longitudinal direction. As a result, the well-developed oriented crystals may increase the diffraction intensity of the annealed calendered samples. Furthermore, the orientation of a part of crystals in a longitudinal direction is conducive to enhancement in the tensile yield strength and modulus of the annealed calendered samples.

#### 3.2.2. Microscopic Morphology Analysis 

**Figure 12:**
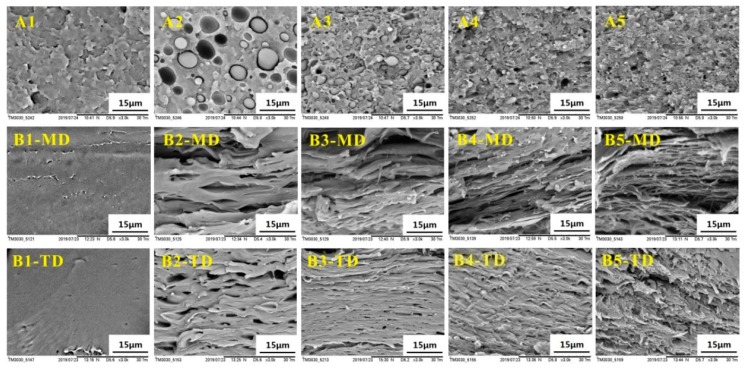
SEM images of annealed PLLA and PLLA/EPDM/OMMT (90/10/*x*) samples prepared by (**A**) compression-molding/(**B**) calendering (**1**-Neat PLLA, **2**-90/10, **3**-90/10/1, **4**-90/10/2, and **5**-90/10/4).

In order to study the morphologies of the annealed PLLA/EPDM/OMMT samples, the cryo-fractured surfaces of the annealed samples were observed by SEM, and their SEM images are presented in Figure 12. Clearly, the typical droplet morphology is still maintained in the annealed compression-molded PLLA/EPDM/OMMT samples, similar to the morphology of the compression-molded ones without annealing (see Figure 3). With increasing OMMT loading, the size of EPDM particles also decreases drastically. Moreover, similar to the calendered samples without annealing (see Figure 3), the annealed calendered ones still show a strongly oriented morphology, in which amount of pre-stretched EPDM phases disperse uniformly in longitudinal direction. This indicates that the morphologies of the EPDM dispersed phases can remain almost unchanged, although the samples have been annealed and fully crystallized under the given annealing condition. 

#### 3.2.3. Tensile Performance

**Figure 13:**
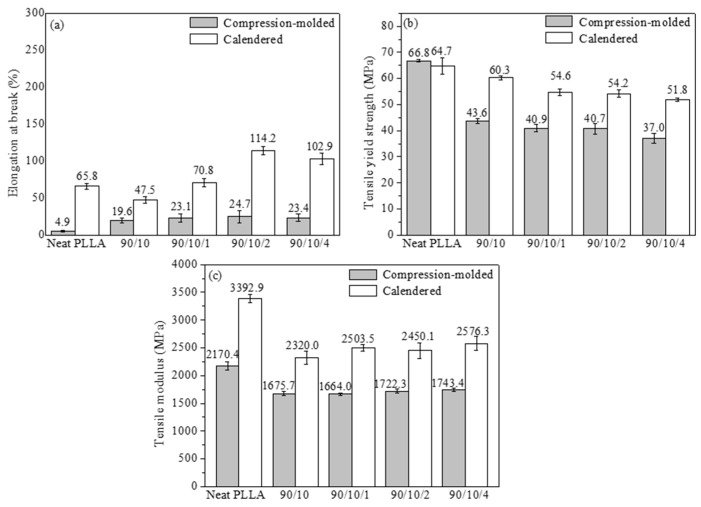
(**a**) elongation at break; (**b**) tensile yield strength; and (**c**) tensile modulus of annealed PLLA and PLLA/EPDM/OMMT (90/10/x) samples prepared by compression-molding and calendering.

The elongation at break, tensile yield strength, and tensile modulus of the annealed samples are presented in Figure 13. Figure S4 shows the tensile stress–strain curves of all the annealed samples. Similar to the compression-molded PLLA sample without annealing, the annealed compression-molded one also shows a classic brittle fracture behavior (*ε* = 4.9%). Moreover, *ε* of the annealed calendered PLLA sample decreases from 108.9% for the calendered PLLA one without annealing to 65.8%. While the samples were annealed and fully crystallized, the formed crystals would contact and even squeeze each other to construct a 3D physical crosslinking structure in the PLLA matrix, which could not only weaken the yielding ability of the PLLA matrix, but also accelerate the craze-to-microcrack transition. With the addition of 10 wt. % EPDM, *ε* of the annealed compression-molded PLLA/EPDM (90/10) sample increases to only 19.6%. Furthermore, with the incorporation of 1 phr–4 phr OMMT, the annealed compression-molded ternary samples show only a slight increase in tensile ductility, and *ε* ranges between 23.1–24.7%. It can be pointed out that the compatibilizing effect of OMMT on the PLLA/EPDM samples is inhibited seriously for the annealed samples. This can be attributed to the following facts: the formed physical crosslinking crystalline structure in the annealed blending samples weakens the yielding ability of the blending samples. Moreover, more crazes are initiated and a crazes-to-microcracks transition is more easily achieved. In this case, the spherical/ellipsoidal EPDM particles show a relatively weak ability to terminate craze, resulting in a premature fracture behavior in the annealed blending samples. For the annealed calendered PLLA/EPDM/OMMT (90/10/x) samples, with increasing OMMT loading from zero to 1, 2, and 4 phr, *ε* increases from 47.5% to 70.8%, 114.2%, and 102.9%, respectively. This can be attributed to the fact that the pre-stretched EPDM phase with reduced dimension has a stronger ability to terminate craze, and can improve the yielding ability of the matrix as well. Moreover, compared to the compression-molded samples without annealing, the annealed ones have no obvious increase in tensile yield strength and modulus. However, the tensile yield strength and modulus of the calendered samples increased to an extent after annealing at 80 °C for 45 min. For the annealed calendered samples, XRD patterns confirmed that there should exist some pre-oriented crystals in longitudinal direction, which transformed from the pre-oriented RAF and the tiny crystalline grains after being annealed. Compared to the calendered samples with low crystallinity, the pre-oriented crystalline structure of the annealed calendered samples would further enhance the tensile yield strength and modulus. 

As a result, a series of PLLA/EPDM/OMMT samples with excellent tensile performance and high crystallinity was successfully prepared by calendering and annealing.

## 4. Conclusions

PLLA/EPDM samples with various amounts of OMMT were melt-compounded and then processed into sheets via two routes (compression-molding and calendering). Tensile performance, morphology, and thermal property of all the samples were investigated. Tensile test shows that the incorporation of OMMT resulted in significant enhancement in the tensile ductility of the PLLA/EPDM samples. SEM observation shows that the EPDM domain size decreases largely with increasing OMMT loading, indicating that OMMT has a compatibilization effect on PLLA/EPDM blends. Moreover, the elongation at break, tensile yield strength, and modulus of the calendered samples are much higher than those of the compression-molded samples—in particular for the PLLA/EPDM/OMMT (90/10/2) and (90/10/4) calendered samples. This can be attributed to the pre-oriented RAF of PLLA matrix and pre-stretched EPDM phases in the calendered samples produced by the stretching/shearing effect of calendering. Compared to the spherical/ellipsoidal EPDM particles in the compression-molded samples, an amount of stretched EPDM phases appeared in the calendered samples. These stretched EPDM phases with a higher aspect ratio can be more effective to initiate craze. Moreover, they can also be more effective to terminate the craze growing into a crack along the transversal direction. Therefore, the calendered samples exhibited a better tensile ductility than the compression-molded ones. It is clear that the compatibilizer OMMT and stretching/shearing effect of calendering can effectively tailor the morphology of EPDM phase, and then significantly improve the tensile performance of the PLLA/EPDM sample. Moreover, annealing was carried out to increase the crystallinity of the samples. Owing to a physical crosslinked crystalline structure formed in the annealed samples, the ductility of the annealed samples decreased. However, the calendered samples still maintained a relatively high elongation at break than the compression-molded samples. SEM observation showed that the pre-stretched EPDM phases were maintained in the annealed calendered samples. Further XRD measurement indicated that a pre-oriented crystalline structure was formed in the annealed calendered samples. Owing to these pre-oriented structures, the annealed calendered samples exhibited a better tensile performance than the annealed compression-molded ones.

## Supplementary Materials

The following are available online at https://www.mdpi.com/2073-4360/12/1/106/s1. Figure S1: Tensile stress–strain curves of PLLA and PLLA/EPDM/OMMT (90/10/*x*) samples prepared by (a) compression-molding and (b) calendering. Figure S2: SEM images of cyclohexane-etch cryogenically fracture surfaces and EPDM particle diameter distribution of PLLA/EPDM/OMMT (90/10/*x*) samples prepared by compression-molding (A2-90/10, A3-90/10/1, A4-90/10/2, A5-90/10/4). Figure S3: SEM images of cyclohexane-etch cryogenically fracture surfaces of PLLA/EPDM/OMMT (90/10/*x*) samples prepared by calendering (B2-90/10, B3-90/10/1, B4-90/10/2, B5-90/10/4). Figure S4: Tensile stress–strain curves of annealed PLLA and PLLA/EPDM/OMMT (90/10/*x*) samples prepared by (a) compression-molding and (b) calendering. Table S1: DSC thermodynamic parameters of PLLA and PLLA/EPDM/OMMT (90/10/*x*) samples prepared by compression-molding and calendering. Table S2: DSC thermodynamic parameters of annealed PLLA and PLLA/EPDM/OMMT (90/10/*x*) samples prepared by compression-molding and calendering.
