# Supplementary material for: Effects of Organic Montmorillonite (OMMT) and Pre-Orientation on Property of Poly(l-lactic acid) (PLLA)/Ethylene Propylene Diene Monomer (EPDM) Blends
Source: Polymers (Basel). 2020 Jan 4;12(1):106. doi: 10.3390/polym12010106 (PMC7023235; doi:10.3390/polym12010106)
Supplement: Supplementary file 1 [file polymers-12-00106-s001.pdf]

polymers

MDPI

Supplementary Materials

Effects of organic montmorillonite (OMMT) and pre-orientation on property of Poly(L-lactic acid) (PLLA)/ethylene propylene diene monomer (EPDM) blends

Di Song [1,4], Kai Wang [1,4], Jianing Shen [1,4], Long Zhao [1,4], Nai Xu [1,4,*], Sujuan Pang [2,4] and Lisha Pan [3,4]

[1] School of Materials Science and Engineering, Hainan University, Haikou 570228, China; songdi716@163.com (D.S.); wang_kai126@163.com (K.W.); shenjianing0823@163.com (J.S.); m18334680283@163.com (L.Z.)
[2] School of Science, Hainan University, Haikou 570228, China; psjuan@hainanu.edu.cn (S.P)
[3] School of Chemical Engineering and Technology, Hainan University, Haikou 570228, China; happylisap@163.com (L.P.)
[4] Hainan Provincial Fine Chemical Engineering Research Center, Hainan University, Haikou 570228, China
* Correspondence: xunai@hainanu.edu.cn (N.X.); Tel.: +86-1313-602-3445 (N.X.)

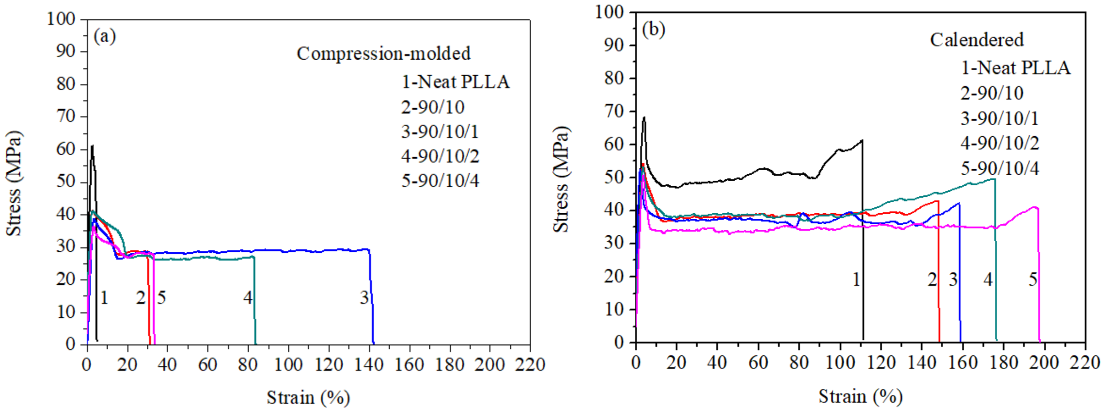

Figure 1. Tensile stress-strain curves of PLLA and PLLA/EPDM/OMMT (90/10/x) samples prepared by (a) compression-molding and (b) calendering.

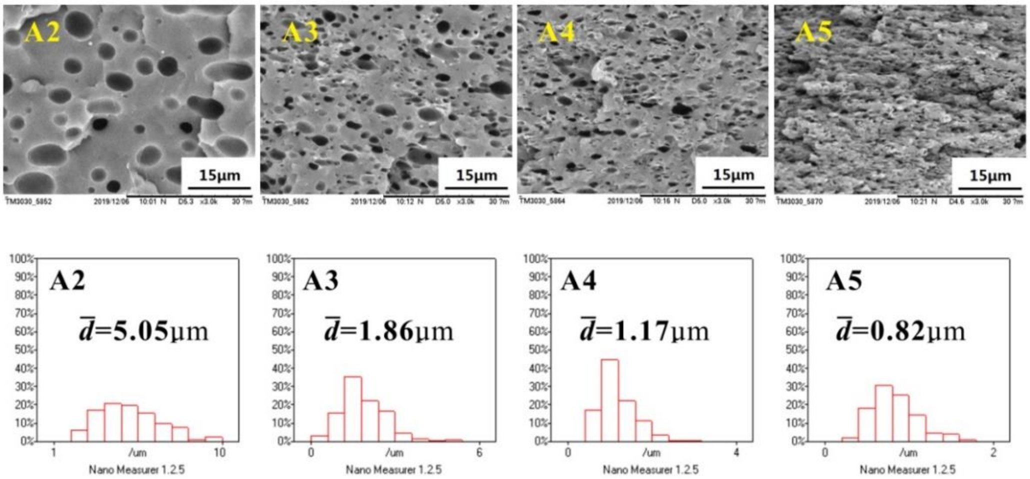

Figure 2. SEM images of cyclohexane-etch cryogenically fracture surfaces and EPDM particle diameter distribution of PLLA/EPDM/OMMT (90/10/x) samples prepared by compression-molding (A**2**-90/10, A**3**-90/10/1, A**4**-90/10/2, A**5**-90/10/4).

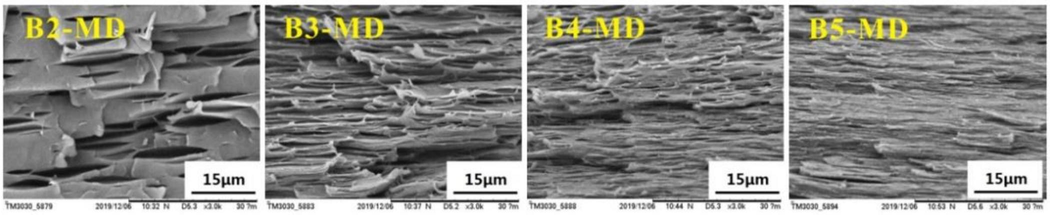

Figure S3. SEM images of cyclohexane-etch cryogenically fracture surfaces of PLLA/EPDM/OMMT (90/10/x) samples prepared by calendering (B**2**-90/10, B**3**-90/10/1, B**4**-90/10/2, B**5**-90/10/4).

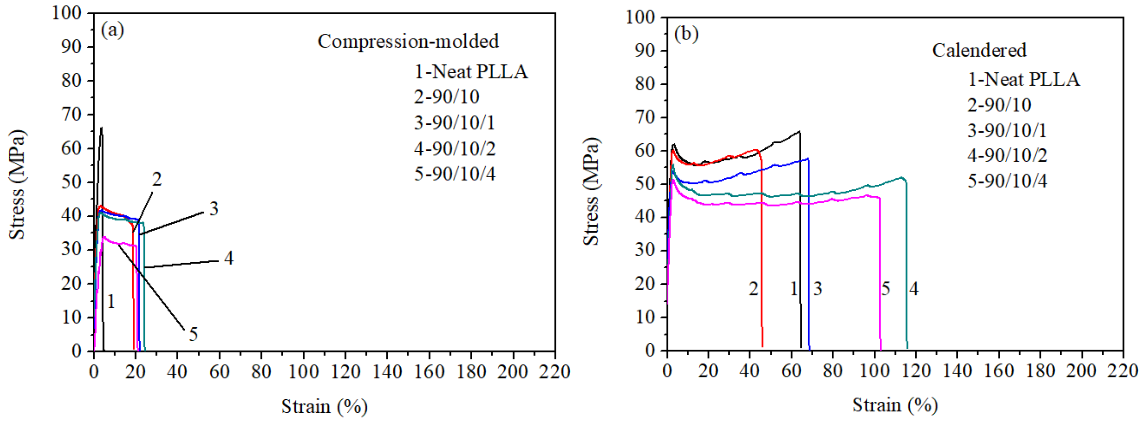

Figure 4. Tensile stress-strain curves of annealed PLLA and PLLA/EPDM/OMMT (90/10/x) samples prepared by (a) compression-molding and (b) calendering.

Table 1. DSC thermodynamic parameters of PLLA and PLLA/EPDM/OMMT (90/10/x) samples prepared by compression-molding and calendering.

	**Samples**	T_{cc} (°C)	T^*_{cc} (°C)	T_m (°C)	ΔH_{cc} (J/g)	ΔH^*_{cc} (J/g)	ΔH_m (J/g)	X_c %
Compression Molding	Neat PLLA	101.2	—	167.7	31.20	—	−31.72	0.55
	90/10	101.7	—	167.2	27.67	—	−28.11	0.52
	90/10/1	103.5	—	167.3	28.52	—	−28.87	0.42
	90/10/2	100.9	—	167.1	27.93	—	−28.15	0.27
	90/10/4	103.0	—	167.6	28.11	—	−28.26	0.18
Calendering	Neat PLLA	81.1/98.8	154.7	167.0	10.33/14.42	0.29	−35.90	11.59
	90/10	88.7/98.3	154.6	166.8	11.65/10.94	0.98	−32.16	10.18
	90/10/1	80.8	153.5	166.4	22.49	1.01	−31.71	9.83
	90/10/2	84.7	152.7	166.5	24.50	1.45	−32.02	7.34
	90/10/4	91.5	153.2	166.4	26.43	1.63	−31.42	4.14

Table 2. DSC thermodynamic parameters of annealed PLLA and PLLA/EPDM/OMMT (90/10/x) samples prepared by compression-molding and calendering.

	**Annealed Samples**	T^*_{cc} (°C)	T_m (°C)	ΔH^*_{cc} (J/g)	ΔH_m (J/g)	X_c %
Compression Molding	Neat PLLA	152.5	166.6	3.80	−37.77	36.25
	90/10	151.0	166.2	3.90	−33.17	34.71
	90/10/1	152.8	166.9	4.58	−33.07	34.12
	90/10/2	152.5	166.8	4.97	−32.84	33.71
	90/10/4	149.8	166.1	5.15	−33.55	35.02
Calendering	Neat PLLA	152.6	166.9	1.96	−38.40	38.89
	90/10	152.8	167.1	2.29	−32.39	35.69
	90/10/1	152.6	166.7	2.63	−32.65	35.95
	90/10/2	152.7	166.7	3.01	−32.95	36.21
	90/10/4	152.3	166.5	3.11	−32.98	36.84
